# Storage conditions and antiviral efficacy of yeast-derived vacuoles on T4 virus

**DOI:** 10.1128/spectrum.02886-25

**Published:** 2026-02-26

**Authors:** Taehwan Kim, Jae-Hwang Jeong, Yang-Hoon Kim, Jiho Min

**Affiliations:** 1 Graduate School of Semiconductor and Chemical Engineering, Jeonbuk National University 26714, Jeonju-si, Jeonbuk, Republic of Korea; 2 Research Division for Biotechnology, Advanced Radiation Technology Institute, Korea Atomic Energy Research Institute (KAERI) 65405, Jeongeup, Jeonbuk, Republic of Korea; 3 Biopharmaceutical Engineering, Chungbuk Provincial University 226508, Cheongju, Chungbuk, Republic of Korea; 4 School of Biological Science, Chungbuk National University 34933, Cheongju, Chungbuk, Republic of Korea; Public Health Agency of Canada, Winnipeg, Manitoba, Canada

**Keywords:** yeast-derived vacuoles, T4 virus, non-enveloped tailed virus, antiviral effect, storage condition

## Abstract

In response to the urgent need for effective antiviral agents, this study explores the potential of vacuoles isolated from yeast in combating non-enveloped tailed viruses, using the T4 virus as a model. Concentration- and time-dependent assays revealed that vacuoles significantly inhibit T4 virus infectivity, achieving over 80% inhibition at 250 μg/mL. Morphological analysis via Bio-TEM imaging unveiled structural changes in the T4 virus after vacuole treatment, including separation of the capsid and tail, leading to impaired virion integrity. Optimization of vacuole storage conditions, particularly storing vacuoles in a pellet state, enhanced their antiviral efficiency. Characterization studies revealed structural modifications in vacuoles stored in the pellet state, such as increased particle size and changes in surface charge properties, potentially facilitating increased interaction with virion particles. These findings underline the promising potential of yeast-derived vacuoles as eco-friendly and effective antiviral agents against non-enveloped tailed viruses and provide insights into their mechanism of action. Further research is needed to elucidate molecular-level interactions and evaluate efficiency against other non-enveloped viruses. By offering novel insights into the antiviral potential of vacuoles, this study contributes to the development of eco-friendly antiviral strategies to address global health challenges.

**IMPORTANCE:**

Non-enveloped viruses remain difficult to inactivate without harsh chemicals or heat. This study introduces yeast-derived vacuoles as a biologically based antiviral platform that disables a model non-enveloped bacteriophage (T4) with >80% inhibition at 250 μg/mL. Bio‑TEM reveals capsid-tail disassembly after vacuole exposure, linking macroscopic loss of infectivity to a defined structural mechanism. Storage engineering—maintaining vacuoles in pellet form—enhances efficacy and correlates with increased particle size and altered surface charge, suggesting tunable physicochemical interactions with virions. These results establish vacuoles as scalable, eco-friendly antiviral agents and provide design rules (dose, contact time, storage state, surface properties) for optimizing activity. Because the approach targets virion integrity rather than specific proteins, it may generalize across non-enveloped viruses, motivating molecular-level studies and translational testing. The work broadens the antiviral toolkit by leveraging a safe, low-cost cellular organelle.

## INTRODUCTION

Viruses are critical pathogens in public health, responsible for food poisoning, waterborne diseases, and infectious diseases such as COVID-19 (SARS-CoV-2), which caused the 2019 pandemic (1–3). Various efforts have been made to inactivate infectious virions in different sample types (4–8). However, chemical antiviral agents have significant limitations. Common chemicals like hypochlorous acid (HClO) and sodium hypochlorite (NaClO) can irritate the respiratory and digestive tract mucosa and threaten the surrounding ecosystem if used in large quantities (9, 10). Developing low-risk, non-toxic, and effective disinfectants is essential to avoid environmental impact and preserve biodiversity (10).

Non-enveloped viruses have a capsid that protects the viral genome and ensures its delivery into host cells during infection. Capsids are highly stable, allowing survival in harsh environmental conditions and ensuring genome delivery for replication and translation within host cells (11, 12). While non-enveloped viruses consist only of a capsid for protection and genome delivery, some viruses, such as bacteriophages, also possess a tail structure that facilitates host recognition and genome injection. *Escherichia* virus T4 (Bacteriophage T4, Phage T4, T4 virus) is a representative non-enveloped virus used as a model agent in research due to its safety, as it is non-pathogenic to humans or eukaryotic cells (13). T4 virus, from the family Myoviridae, infects *Escherichia coli* by injecting its genome using a contractile injection mechanism. It is a relatively large virus, with a width of approximately 90 nm and a length of 200 nm. Its double-stranded DNA (dsDNA) genome is about 168.9 kbp long and encodes 289 proteins (14). Upon infection at 37°C, the lytic infection cycle results in bacterial destruction within 25 min, releasing approximately 300 new progeny virions per infected cell (15–17).

Vacuoles are intracellular organelles in eukaryotes containing weakly acidic hydrolases that decompose and digest waste products (18). Vacuolar enzymes, including proteases, glycolytic enzymes, and lipases, maintain physiological homeostasis by degrading extravacuolar macromolecules involved in intermediary metabolism (19). It is possible that vacuolar enzymes can decompose invading bacteria or viruses (8). However, no studies have investigated the antiviral effects of vacuoles on non-enveloped tailed viruses.

This study aims to confirm the antiviral activity of eco-friendly vacuoles isolated from yeast (*Saccharomyces cerevisiae*) against non-enveloped tailed viruses and identify conditions that enhance this activity. We focus on the potential of vacuoles as antiviral agents beyond non-enveloped tailed viruses. Using vacuoles isolated from yeast, we evaluated their ability to inactivate non-enveloped tailed viruses as an alternative to chemical antiviral agents. Plaque analysis was conducted to measure the reduction in infectivity due to vacuole treatment, and transmission electron microscopy (TEM) imaging was used to observe structural destruction of the T4 virus. Additionally, we examined how storage conditions affect the antiviral activity of yeast-derived vacuoles.

## MATERIALS AND METHODS

### Bacterial host and T4 virus amplification

*E. coli* (BL21 (DE3)) from Novagen (Merck, Germany) was used for phage propagation. The bacteria were cultured in Lysogeny Broth (LB) medium (1% Bacto-Tryptone, 0.5% yeast extract, 1% NaCl). The *Escherichia* virus T4 (T4 virus, BP-5204) was obtained from the Bacteriophage Bank of Korea (LyseNTech, Korea) and maintained in SM Phage Buffer (0.1 M NaCl, 8 mM MgSO_4_, 50 mM Tris-HCl, pH 7.5).

To amplify the T4 virus, *E. coli* was first cultured in 10 mL LB medium at 37°C, 180 rpm for 20 h. For the main culture, 100 μL of this seed culture was added to 10 mL LB medium and incubated at 37°C, 180 rpm for 3.5 h until the optical density at 600 nm (OD_600_) reached 0.7. Next, 200 μL of *E. coli* was mixed with 5 mL of 40°C Top Agar (1% Bacto-Tryptone, 0.5% yeast extract, 1% NaCl, 0.7% Bacto-Agar) and poured onto an LB plate (Bottom Agar; 1% Bacto-Tryptone, 0.5% yeast extract, 1% NaCl, 1.5% Bacto-Agar). After solidification for 30 min, the plates were incubated at 37°C for 2 h to allow host cell growth. Then, 200 μL of T4 virus was spread on the solidified LB plate and incubated overnight at 37°C. The top agar layer, where the virus amplified and cleared, was collected using a cell scraper and eluted in 5 mL of SM Phage Buffer on a rocking shaker for 2 h. The mixture was centrifuged at 3,500 rpm for 10 min to remove the agar, and the supernatant was filtered through a 0.2 μm syringe filter. Both the T4 virus and *E. coli* were stored at −70°C in 15% glycerol in SM Phage Buffer and LB, respectively.

### Plaque assay

The recovered virus supernatant was prepared by serial dilution 10 times using LB medium and used to infect host cells (*E. coli*). *E. coli* was pre-inoculated in 10 mL LB medium in a conical tube and incubated with shaking at 180 rpm at 37°C for 20 h. One hundred μL of *E. coli* (seed culture; cultured in advance for 20 h) was used for the main culture in 10 mL of LB medium, and cultured with shaking in a conical tube at 180 rpm at 37°C for 3.5 h until the optical density at 600 nm (OD_600_) reached 0.7. Two hundred μL of *E. coli* was dispensed into a microtube, and 10 μL of diluted virus supernatant was added and incubated for 5 min. The infected host cell-virus mixture was transferred to a conical tube containing 5 mL of Top Agar at approximately 40°C, mixed briefly, and then poured onto a pre-heated LB plate. After standing for 30 min to harden, the plates were incubated overnight at 37°C. The virus titer was determined by counting transparent plaques.

### Isolation of vacuoles from *S. cerevisiae*

Yeast-derived vacuoles were used to examine antiviral effects. *S. cerevisiae* (BJ3501; ATCC208280) was provided by the Korea Research Institute of Bioscience and Biotechnology (KRIBB, Korea) and propagated in YPD medium (1% yeast extract, 2% peptone, 2% D-glucose). For the seed culture, *S. cerevisiae* was incubated in 10 mL YPD medium at 180 rpm and 30°C for 24 h. When OD_600_ exceeded 0.6, 1% (vol/vol) of the seed culture was used to inoculate 1 L of YPD medium in an Erlenmeyer flask, incubated at 180 rpm and 30°C for 24 h. Yeast cells in the exponential phase (OD_600_ of 0.8–0.9) were harvested by centrifugation at 3,000 rpm for 5 min and washed twice with sterilized distilled water (dH_2_O). The final cell yield was approximately 8 g/L. This process was repeated to obtain sufficient quantities of yeast cells.

For vacuole extraction, 5 g of yeast cells was mixed with 25 mL of 0.1 M Tris-SO_4_ buffer (pH 9.4) containing 10 mM dithiothreitol (DTT) and incubated at 90 rpm and 30°C for 15 min to weaken the cell walls. After chilling on ice for 5 min and centrifuging at 3,000 rpm for 5 min to remove the supernatant, 5 g of glass beads was added to the cell mixture in a 1:1 mass ratio. The cells were resuspended in 20 mL of breaking buffer (20 mM Tris-HCl [pH 7.4], 0.6 M sorbitol) and 200 μL of 0.1 M PMSF (final concentration 1 mM), then vortexed intermittently on ice for 20 min. The mixture was centrifuged at 500 × *g* for 5 min, and the supernatant was collected and re-centrifuged at 20,000 × *g* and 4°C for 30 min. The resulting pellet (vacuoles) was washed twice with sterilized dH_2_O and re-centrifuged at 20,000 × *g* for 5 min (18).

To quantify the concentration of yeast-derived vacuoles, soluble proteins were extracted from the vacuoles through lysis. A small amount of the pellet was mixed with lysis buffer (0.1% NP-40, 5 mM DTT, and 0.1 mM PMSF) at a 1:1 volume ratio and chilled on ice for 5 min. The mixture was vortexed intermittently for 20 min, chilled on ice for 30 min, and centrifuged at 13,000 rpm and 4°C for 10 min. The amount of soluble protein in the supernatant was measured using a Bradford assay (Bio-Rad, USA) to estimate the concentration of vacuoles.

### Storage of vacuoles from *S. cerevisiae*

To improve antiviral activity, the storage conditions of vacuoles extracted from yeast were varied. The extracted vacuoles were mixed with sterilized dH_2_O in a 1:1 volume ratio, stored at 4°C, and used as soon as possible (original method; recommended for use within 2 weeks). Alternatively, vacuoles were stored in the pellet state by removing the supernatant (dH_2_O) through centrifugation at 20,000 × *g* for 5 min and then stored at 4°C for 10 days (different storage conditions). Before use in all experiments, each vacuole preparation was centrifuged to remove as much supernatant as possible, and the concentration of the vacuoles was determined using the method described in “Isolation of vacuoles from *S. cerevisiae*,” above.

### Treatment of yeast-derived vacuoles and T4 virus analysis

Infection inhibition against the T4 virus was confirmed depending on the treatment concentration and treatment time of the extracted vacuoles. T4 virus (5 × 10^5^ pfu/mL) was treated with soluble protein-containing vacuoles at each concentration (25, 50, 100, and 250 μg/mL). Vacuoles were treated at room temperature (RT) for each time interval (15 min, 30 min) to determine the antiviral effect depending on treatment time and concentration. Distilled water was set as a control to confirm the infection-inhibiting effect of the vacuoles. The vacuole-treated virus mixture was centrifuged at 20,000 × *g* for 5 min to recover only the supernatant. Based on the recovered supernatant, the virus titer was determined using a plaque assay, and morphological analysis was performed using TEM.

To evaluate the antiviral activity of vacuoles, the viral titer was measured using a plaque assay to accurately determine the concentration of infectious units, described in “Plaque assay,” above. The recovered virus supernatant, whose titer was determined, was diluted to 5 × 10^5^ pfu/mL with SM Phage Buffer and treated with soluble protein-containing vacuoles at each concentration (25, 50, 100, and 250 μg/mL) diluted with dH_2_O. To confirm the infection inhibition effect of vacuoles according to each treatment time and concentration at RT, the virus titer was measured by checking the number of transparent plaques by the method described in “Plaque assay,” above, after vacuole treatment. Results are expressed as infection inhibition percentage (%).

### Microscopic analysis (Bio-TEM) for morphology investigation of T4 virus and vacuoles

Non-enveloped tailed viruses have a capsid that protects the genome and a contractile tail that facilitates the entry of the genome into the host cell. The morphology of the vacuole-treated T4 virus and the isolated vacuoles was confirmed by Bio-TEM. T4 virus (1.0 × 10^10^ pfu/mL) was treated with soluble protein-containing vacuoles at 20 μg/mL. The vacuole-virus mixture was incubated at 20 rpm at RT using a laboratory rotator for 3 h. The mixture was serially diluted 10-fold using sterilized and filtered distilled water and used to obtain TEM images. Ten μL of the diluted mixture was placed on a carbon film 400 mesh-Cu (Cat#CF400-CU; Electron Microscopy Sciences, USA). After 15 min of drying to settle the sample particles into the grid, the mixture was negatively stained with 1% uranyl acetate for a few seconds. Residual uranyl acetate was carefully removed, and the sample was dried for 10 min to ensure complete dryness. TEM images were obtained with a Hitachi H-7650 transmission electron microscope (Japan) installed in the Center for University-Wide Research Facilities (CURF) at Jeonbuk National University. The Bio-TEM was operated/aligned at 100 kV, and images were obtained at magnifications of ×100,000.

### Characterization analysis of vacuoles

Characteristic analysis was performed using field emission scanning electron microscope (FE-SEM), dynamic light scattering (DLS), and electrophoretic light scattering (ELS) to confirm the morphological changes and structural stability of vacuoles according to changes in storage conditions. Then, pH analysis was performed using an Orion Star A211 Advanced pH Benchtop Meter (Thermo Fisher Scientific, Massachusetts, USA).

#### Microscopic analysis for the morphology investigation of vacuoles

FE-SEM analysis was performed to obtain three-dimensional images of vacuoles and analyze their morphologies. The vacuoles were resuspended in dH_2_O, stored in a deep-freezer at −70°C overnight, and then lyophilized in a speed vacuum concentrator (Modulspin 40 and ColdVac 80; Biotron, Inc., Gyeonggi-do, Korea) at −85°C for 3 h. All samples were prepared by lyophilization into a complete powder form. The dried powder was fixed on carbon tape on a specimen stage and coated with platinum (Pt) for 45 s under vacuum conditions (4.0 × 10^−2^ mbar) using a Leica EM ACE200 Low Vacuum Coater (Leica Microsystems CMS GmbH, Wetzlar, Germany). FE-SEM images of coated samples were obtained with a Zeiss Supra 40VP scanning electron microscope (Carl Zeiss, Oberkochen, Germany) installed in the Center for University-Wide Research Facilities (CURF) at Jeonbuk National University. The FE-SEM was operated/aligned at 2 kV, and images were obtained at magnifications of ×25,000.

#### DLS analysis to investigate the size distribution of vacuoles

Nanoparticle size analysis using the DLS method (also called the photon correlation method) was employed to analyze the distribution of vacuole particle sizes (20). The extracted vacuoles were diluted 1:200,000, and the diluted vacuole solution was injected into an ELSZ neo (Otsuka Electronics, Japan) instrument to analyze the nanoparticle size with a homodyne optical system in the Interventional Mechano-Biotechnology Convergence Research Center.

#### Zeta potential analysis using ELS

Zeta potential analysis using the ELS method (also called the laser Doppler method) was performed to observe electrophoresis according to the charge of vacuole particles by applying an electric field to particles in solution. The extracted vacuoles were diluted 1:200,000, and the diluted vacuole solution was injected into an ELSZ neo (Otsuka Electronics, Japan) instrument to analyze the zeta potential with a heterodyne optical system in the Interventional Mechano-Biotechnology Convergence Research Center. The electrostatic properties and stability of vacuoles stored in the pellet state were characterized by zeta potential measurements.

#### pH measurement of vacuoles

Each vacuole sample was prepared from the same yeast culture. Vacuoles were extracted as described in “Plaque assay,” above, and stored as described in “Isolation of vacuoles from *S. cerevisiae*,” above. Vacuoles were diluted with dH_2_O to a concentration of 10 μg/mL, making a 10 mL volume. The diluted vacuoles were measured at 25°C using an Orion Star A211 Advanced pH Benchtop Meter. Prior to measurement, the pH Benchtop Meter was calibrated using standard buffer solutions at pH 4.0, 7.0, and 10.0. After calibration, the electrode was rinsed twice with dH_2_O and gently blotted dry. Each vacuole sample was measured in triplicate to ensure accuracy, and the average value was calculated.

### Other materials

Conical tubes were provided by SPL (Cat#50050, Korea). Petri dishes were provided by SPL (Cat#10060, Korea). Cell scrapers were provided by SPL Life Sciences (Cat#SPL90030, Korea). Minisart Syringe Filters—NML (SFCA—Surfactant-free Cellulose Acetate) were provided by Sartorius (Cat#S6534-FMOSK, Germany). Erlenmeyer flasks were provided by DongSung Science (Cat#HA.1020D.2000, Korea). Microtubes were provided by Axygen (Cat#MCT-175-C, USA). Quick Start Bradford 1× Dye reagent was provided by Biorad (Cat#500-0205, USA). Nunc MicroWell 96-Well plates were provided by ThermoFisher (Cat#167008, USA).

### Data analysis

For all experiments, each data point was obtained from three independent samples conducted simultaneously for error analysis. The results are shown as average standard deviation or correlation under several experimental conditions. The data were analyzed using SigmaPlot (Systat Software Inc., USA). A *P* value < 0.05 was considered significant.

## RESULTS

### Evaluation of the antiviral activity of yeast-extracted vacuoles against T4 virus

**Fig 1:**
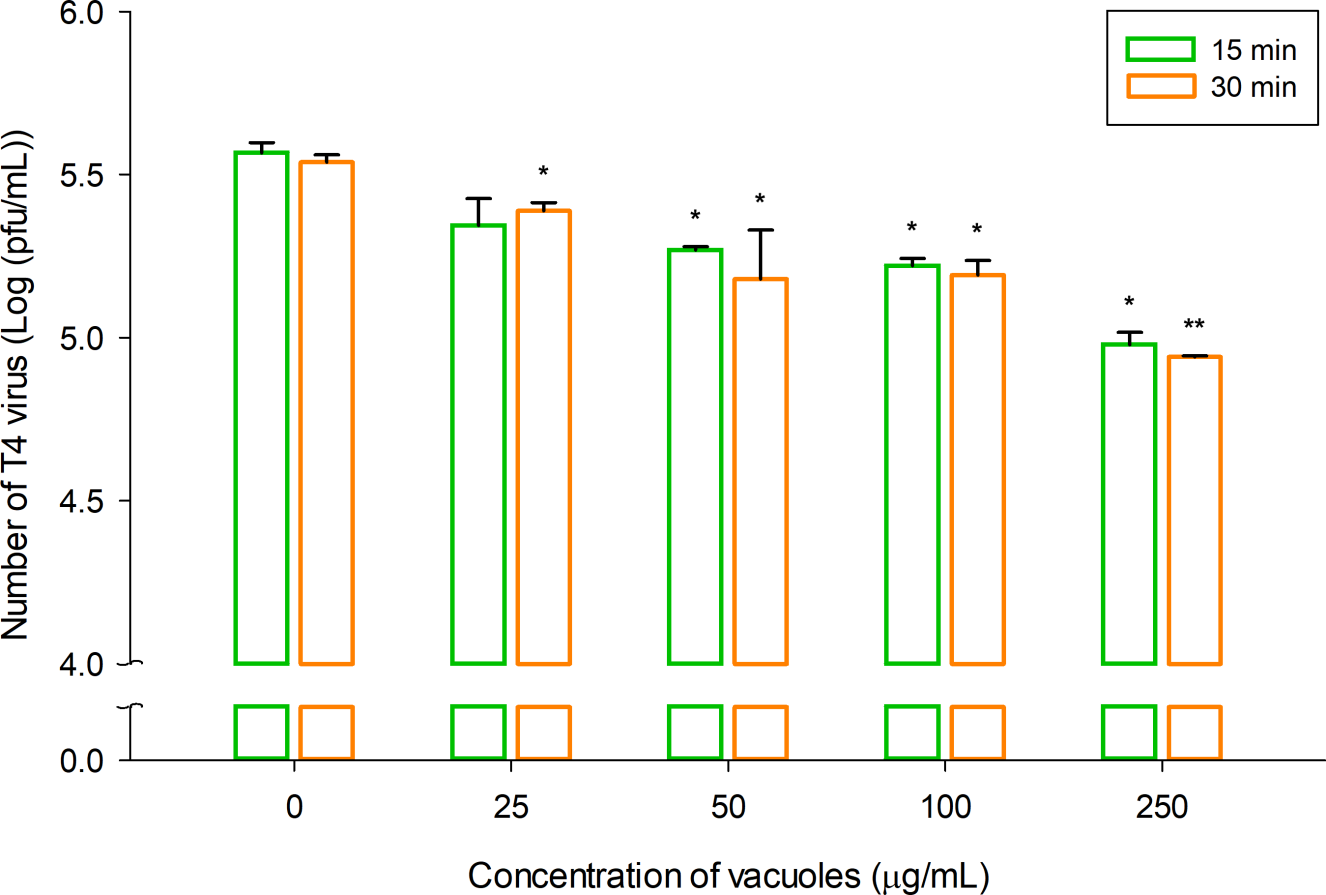
Viral titers of *Escherichia* virus T4 versus vacuole concentration and versus exposure time. Final concentrations were adjusted to 0, 25, 50, 100, and 250 μg/mL using distilled water. Zero (0) is the control group containing only dH_2_O. The data were analyzed using Sigma Plot 14 (Systat Software Inc., USA), and a *P* value < 0.05 was considered significant. Data are expressed as the means of the infection inhibition rate ± SD in %. *P < 0.05 and **P < 0.01 vs. control.

The T4 virus was selected as a surrogate for non-enveloped tailed viruses to evaluate the antiviral activity of vacuoles extracted from yeast. The extracted vacuoles were used as described in “Storage of vacuoles from *S. cerevisiae*,” above. Vacuoles were treated at concentrations of 0 (control; Distilled water), 25, 50, 100, and 250 μg/mL. Antiviral activity was assessed at exposure times of 15 and 30 min using a plaque assay. Treatments were conducted at RT against 5 × 10^5^ pfu/mL of T4 virus. By analyzing the viral titer of T4 virus particles through plaque assays, we confirmed that vacuoles inhibit the infection of the T4 virus, as shown in Fig. 1. Figure 1 illustrates that infectivity decreases with increasing treatment concentration and exposure time. At a treatment time of 15 min, the virus titer was 5.57 ± 0.03 log (pfu/mL) at a control (concentration of 0 μg/mL), which was statistically significantly reduced to 4.98 ± 0.04 log (pfu/mL) at a concentration of 250 μg/mL. This is about 0.59 log (pfu/mL) reduction, which corresponds to about 74.2% reduction efficiency (*P* =0.045). Similarly, at a treatment time of 30 min, the virus titer was 5.54 ± 0.02 log (pfu/mL) at a control (concentration of 0 μg/mL), which was statistically significantly reduced to 4.94 ± 0.00 log (pfu/mL) at a concentration of 250 μg/mL. This is about 0.60 log (pfu/mL) reduction, which corresponds to about 74.7% reduction efficiency (*P* =0.0027). Overall, the virus titer showed an exponential decrease as the treatment concentration increased, indicating a dose-dependent effect. Infection inhibition rates for treatment concentrations of 50 and 250 μg/mL at various exposure times (15, 30, 60, and 120 minutes) are detailed in Table S1. At a concentration of 50 μg/mL, an antiviral effect of over 80% was observed, with a reduction from 5.12 ± 0.08 log (pfu/mL) to 4.88 ± 0.06 log (pfu/mL) after 60 min, and a similar effect was confirmed at 250 μg/mL, with 4.86 ± 0.12 log (pfu/mL) in just 15 min. The data for the 250 μg/mL concentration in Table S1 showed a similar trend to the data presented in Fig. 1.

The extracted vacuoles were used as described in “Storage of vacuoles from *S. cerevisiae*,” above. To check for errors that may occur due to resuspension in dH_2_O and storage at 4°C, the extracted vacuoles were used immediately (within 1 day) to confirm the antiviral effect. Even when the extracted vacuoles were used directly, an infection inhibition rate of approximately 55%–60% was observed (Fig. S1). However, it was confirmed that vacuoles stored at 4°C had a slightly higher antiviral effect at the same treatment concentration than vacuoles used immediately after extraction (not significant). The antiviral effect results obtained under different storage conditions are described in “Effects of storage conditions on vacuole antiviral efficacy,” below.

### Impact of vacuole treatment on T4 virus morphology and infectivity

**Fig 2:**
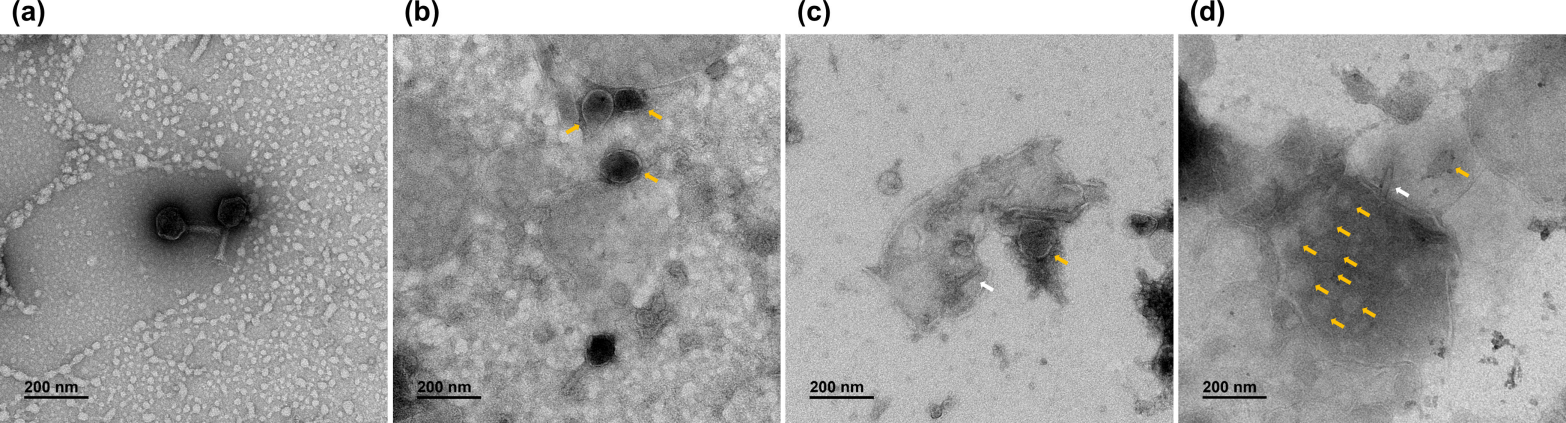
Morphology investigation of *Escherichia* virus T4 and vacuoles using Bio-TEM images. (**a**) Bacteriophage T4 virion and (**b–d**) Bacteriophage T4 treated with vacuoles. White arrows indicate the tail part of phage T4, and yellow arrows indicate the capsid part of phage T4.

To investigate the structural changes in the T4 virus, TEM images were taken after vacuole treatment. The TEM image in Fig. 2a shows the normal virion size and shape of the T4 virus, with a width of about 85 nm (≈850 Å-long) and a length of about 208 nm (≈2075 Å-long), clearly displaying the elongated icosahedral capsid structure and well-contained DNA (21, 22). However, the TEM images post-vacuole treatment (Fig. 2b through d) reveal structural collapse. Notably, the capsid (indicated by yellow arrows) and tail (indicated by white arrows) parts of the T4 viruses appear separated. The separated capsid part displays a spherical rather than an icosahedral shape, and a similar damaged shape within the vacuole is observed, which matches the destructed form of the T4 virus following treatment with other antiviral agents (23). Thus, it was confirmed that vacuole treatment leads to morphological changes in the virion, likely reducing the rate of viral infection by damaging and isolating the tail part essential for infection (24).

### Effects of storage conditions on vacuole antiviral efficacy

To enhance the antiviral activity of vacuoles extracted from yeast, we investigated various storage conditions and identified those that potentially improved antiviral efficacy. Characteristic analysis was conducted to examine the morphological changes and structural stability of vacuoles under different storage conditions.

#### Enhanced antiviral efficacy due to storage conditions

**Fig 3:**
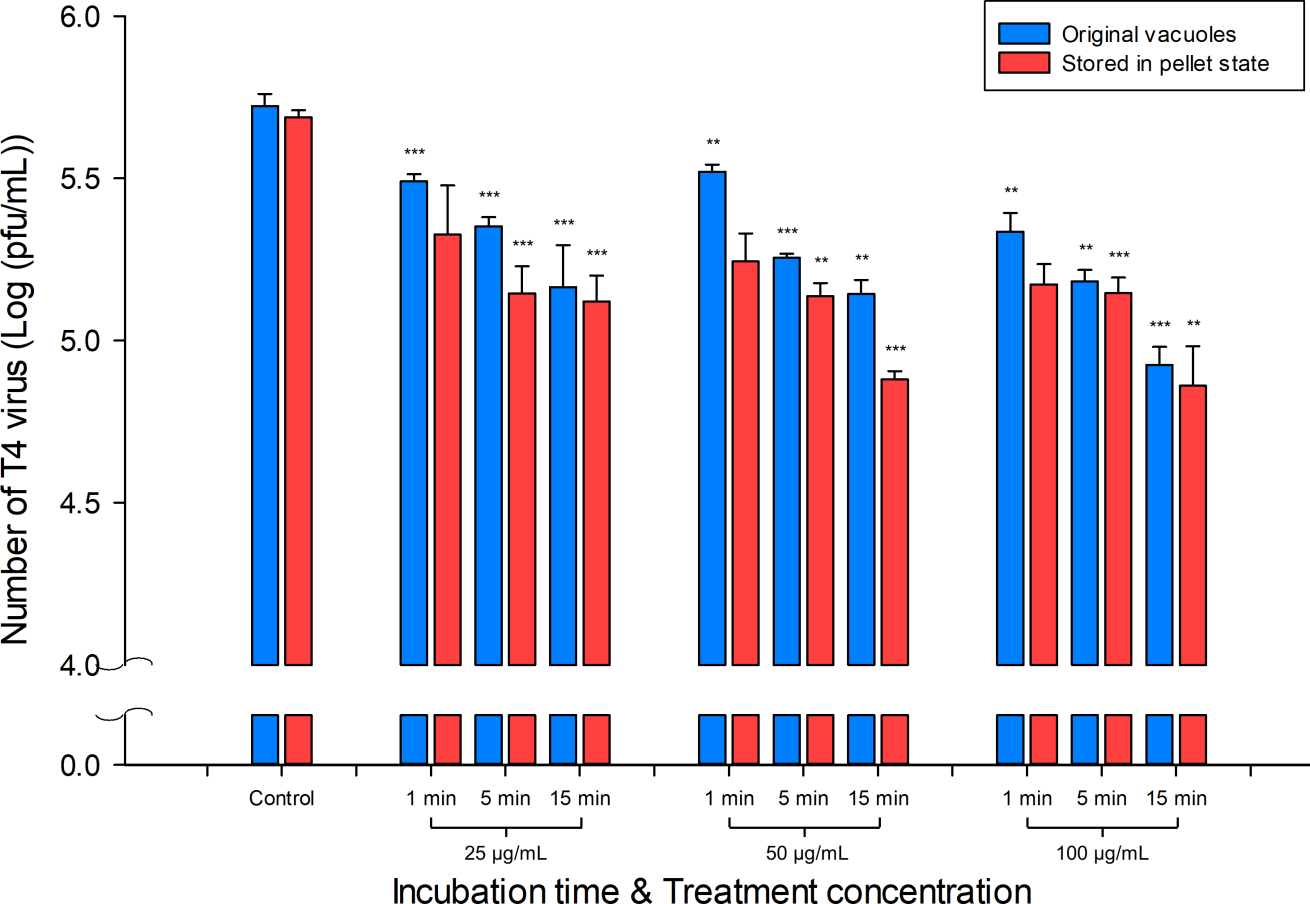
Viral titers (Log_10_ [pfu/mL] ± SD) of *Escherichia* virus T4 versus vacuole stored in pellet state for 10 days. Control refers to the untreated group (incubation time: 0 min, treatment concentration: 0 μg/mL). The data were analyzed using Sigma Plot 14 (Systat Software Inc., USA), and a *P* value < 0.01 was considered significant. Data are expressed as the means of the Log_10_ (pfu/mL) ± SD. **P < 0.01 and ***P < 0.001 vs. control.

To evaluate the antiviral efficacy under different vacuole storage conditions, both vacuoles stored under different conditions and the original vacuoles were treated with 5 × 10^5^ pfu/mL of T4 virus at concentrations of 25, 50, and 100 μg/mL for 1, 5, and 15 min at RT. Plaque assays were used to determine the viral titers, and the results are presented in Fig. 3 on a logarithmic scale. Both treated vacuoles exhibited a reduction in viral titers compared to each control group. At treatment concentrations of 25, 50, and 100 μg/mL, a time-dependent decrease in viral titers was observed. Specifically, for original vacuoles, at a concentration of 25 μg/mL, viral titers decreased from 5.72 ± 0.04 log (pfu/mL) to 5.49 ± 0.02, 5.35 ± 0.03, and 5.16 ± 0.13 log (pfu/mL) after 1, 5, and 15 min of treatment, respectively (paired t-test, *P* =1.0 × 10⁻^4^, 2.8 × 10⁻^9^, and 2.5 × 10⁻^6^). At a concentration of 50 μg/mL, titers decreased to 5.52 ± 0.02, 5.26 ± 0.01, and 5.14 ± 0.04 log (pfu/mL) (*P* = 0.0020, 3.2 × 10⁻⁵, and 0.0038), and at 100 μg/mL, titers further decreased to 5.33 ± 0.06, 5.18 ± 0.04, and 4.93 ± 0.06 log (pfu/mL) (*P* = 0.0038, 0.0011, and 9.6 × 10⁻⁴). For vacuoles stored in pellet state, at a concentration of 25 μg/mL, viral titers decreased from 5.69 ± 0.02 log (pfu/mL) to 5.33 ± 0.15, 5.14 ± 0.08, and 5.12 ± 0.08 log (pfu/mL) after 1, 5, and 15 min of treatment, respectively (paired t-test, *P* =0.1345, 2.7 × 10⁻^4^, and 2.1 × 10⁻^4^). At a concentration of 50 μg/mL, titers decreased to 5.24 ± 0.09, 5.14 ± 0.04, and 4.88 ± 0.03 log (pfu/mL) (*P* = 0.0696, 0.0011, and 1.8 × 10⁻⁴), and at 100 μg/mL, titers further decreased to 5.17 ± 0.06, 5.15 ± 0.05, and 4.86 ± 0.12 log (pfu/mL) (*P* = 0.1424, 2.1 × 10⁻⁴, and 0.0091). Although reductions at 1 min were not always statistically significant, strong and significant antiviral effects emerged at 5 and 15 min, particularly at 50 and 100 μg/mL. To further confirm the antiviral activity, additional experiments were conducted under identical conditions. The enhanced antiviral effect of vacuoles stored in pellet form compared to the original vacuoles is shown in Table S2. Compared to the original vacuoles, vacuoles stored in pellet form had a maximum difference of 28.90%p and 26.80%p higher antiviral effect at 50 μg/mL for exposure times of 1 min and 5 min, and an antiviral effect of approximately 25.37%p higher at 25 μg/mL for an exposure time of 15 min. The data in Fig. 3 and Table S2 indicate that vacuoles stored in the pellet state exhibit a more pronounced reduction in viral titers and enhanced antiviral efficacy compared to the original vacuoles.

#### Characterization analysis of stored vacuoles

**Fig 4:**
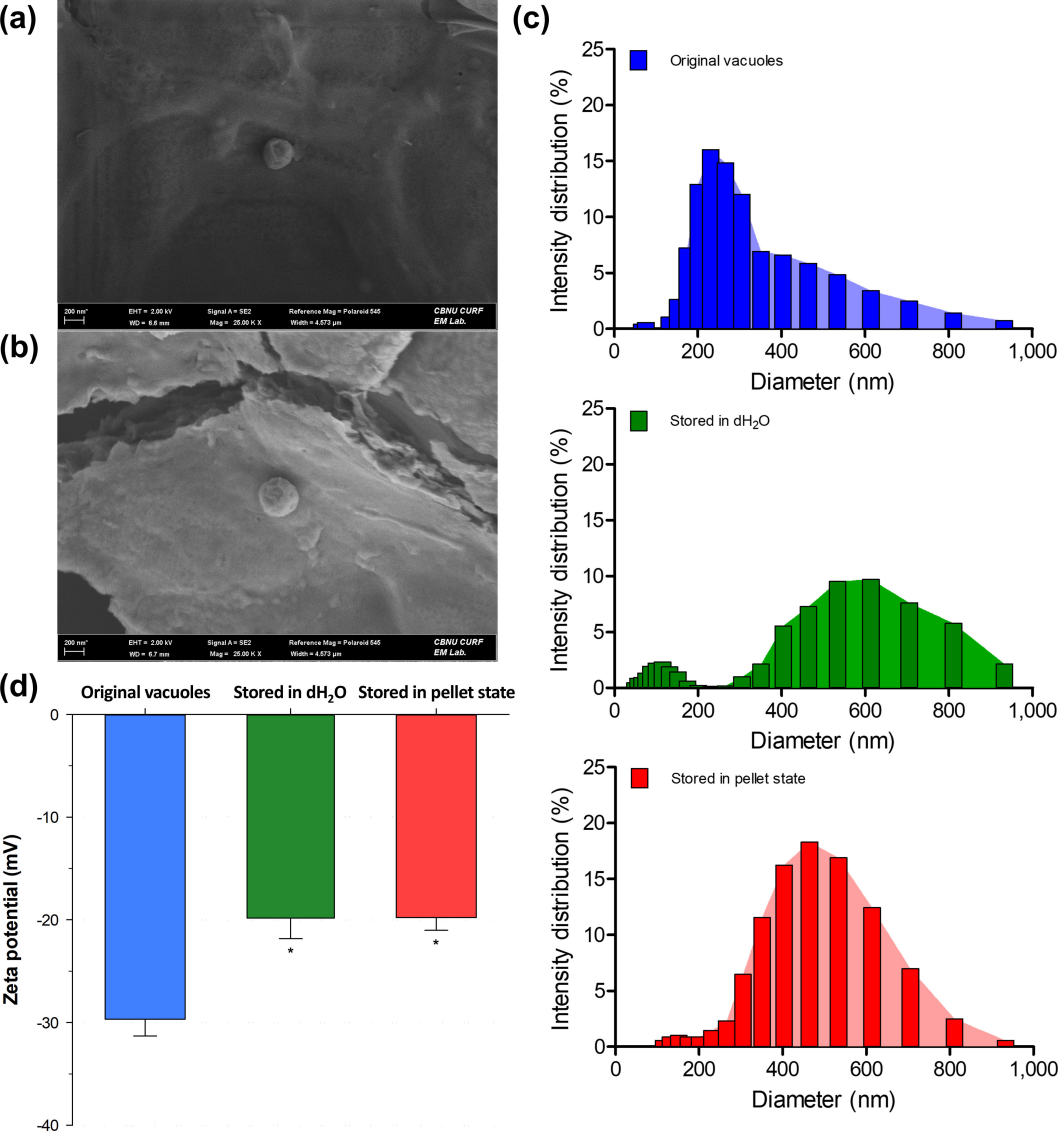
Characteristic analysis of vacuoles. FE-SEM images for morphological analysis of the original vacuoles (**a**) and the vacuoles stored in pellet state (**b**). (**c**) Vacuole particle size distribution determined with DLS. (**d**) Zeta potential determined with ELS. The data were analyzed using Sigma Plot 14 (Systat Software Inc., USA), and a *P* value < 0.05 was considered significant. Data are expressed as the means of the zeta potential of vacuoles ± SD in mV. *P < 0.05 vs. original vacuoles.

Vacuoles stored in the pellet state demonstrated enhanced antiviral efficacy, resulting in viral titers reductions ranging from 70.12% to 72.01% (about 1.89%p; about 2.7% relative reduction) at minimum, and from 39.94% to 68.84% (about 28.90%p; about 72.4% relative reduction) at maximum. To elucidate the enhanced antiviral mechanisms, we performed a detailed characterization of these vacuoles. The morphological characteristics of vacuoles under different storage conditions and the original vacuoles were analyzed using FE-SEM and DLS. The particle size distribution and zeta potential were also measured using DLS and ELS. The vacuoles stored in the pellet state retained their spherical shape, similar to the original vacuoles, but exhibited a slight size reduction (Fig. 4a and b). Specifically, the average diameter of vacuoles stored in the pellet state was increased by 224 nm compared to the original vacuoles (Fig. 4c and Fig. S2). This alteration is likely attributed to the fusion of the vacuolar membrane bilayer (25). The vacuoles dissolved and stored in dH_2_O exhibited the most significant change in diameter, presumably due to a combination of osmotic expansion and membrane bilayer fusion. The size distribution of these vacuoles was broader compared to the original vacuoles. Furthermore, the absolute value of the zeta potential of vacuoles stored in the pellet state was, on average, 9.9 mV lower (Fig. 4d). This reduction in zeta potential indicates structural modifications in the vacuoles, likely altering their surface charge properties. Such changes in charge distribution contribute to a lower absolute value of zeta potential, suggesting that storage in the pellet state can induce changes in the surface charge properties of the particles. The mechanisms underlying the improved antiviral effect of vacuoles are not fully elucidated, so we need to interpret our results with caution. We hypothesize that lower absolute zeta potential values and changes in particle surface charge properties may facilitate the phagocytosis of a greater number of viruses. It should be noted that this hypothesis is based on literature demonstrating that the lower the absolute value of the zeta potential of a nanoparticle, the more non-specific the physical interaction between the phage and the nanoparticle, leading to increased interactions between them (26). Additionally, the pH of the vacuoles stored in pellet state was measured to be approximately 5.84 ± 0.08, while the pH of the original vacuoles was 6.35 ± 0.05. This was measured with vacuole samples obtained from the same yeast culture, and the pH was measured after diluting to a concentration of 10 μg/mL. The observed changes in size, zeta potential, and pH may therefore contribute to the improved antiviral efficacy of vacuoles stored in the pellet state. However, further research is necessary to fully understand these mechanisms.

## DISCUSSION

Antiviral agents have recently been recognized as one of the emerging contaminants in the environment (27). While antibacterial agents have received great attention in recent decades in relation to bacterial resistance, the issues surrounding antiviral agents have only recently begun to be recognized for their potential impact (28–30). Indiscriminate prescriptions and consumption have been made to prevent the spread of viral infectious diseases, and some antiviral agents are emerging as important issues due to the fact that they are not sufficiently removed by existing wastewater treatment methods, and the precise investigation into their effects has not yet been conducted (27). Accordingly, there is an urgent need for eco-friendly and effective antiviral agents with appropriate treatment concentrations that consider various ecosystems (10). As an alternative method, we evaluated yeast-derived vacuoles for application as eco-friendly antiviral agents.

The results presented in this paper demonstrate the potential of yeast-derived vacuoles as effective antiviral agents against non-enveloped tailed viruses, using the T4 virus as a model. Our findings confirm that these vacuoles exhibit preliminary antiviral activity, impacting both the infectivity and morphology of T4 viruses. Furthermore, we investigated the impact of storage conditions on the antiviral effectiveness of vacuoles, revealing insights that enhance their effectiveness. Vacuoles play a role in maintaining homeostasis by breaking down macromolecules through the autophagy process (19). Proteins such as the capsid and tail that make up non-enveloped tailed viruses are important macromolecules responsible for virus survival and infectivity (11, 12). The results of the microscopic morphological analysis (Bio-TEM) confirmed that the vacuole treatment changed/decomposed the protein structure, which plays an important role in T4 viruses. T4 virus has a capsid and contractile tail, whereas, for the vacuoles-treated samples, the capsid and tail parts were separated, leading to structural changes. These morphological changes likely contribute to the inhibition of viral infection, as damage and isolation of the tail region impede its ability to interact with host cells (23, 24, 31). Supporting these results, we observed that vacuole treatment clearly reduces the infectivity of T4 viruses in a concentration/time-dependent manner, with an increased infection inhibition rate. Notably, vacuole treatment at concentrations of 250 μg/mL resulted in over 80% inhibition of viral infectivity, demonstrating the potent antiviral activity of vacuoles against the T4 virus. Combining these results with the mechanism of inducing viral infection by delivering genomes within the capsid into the host cell with a contractile tail demonstrates that the antiviral mechanism of the vacuole can be initiated (14). In addition, the separated capsid did not maintain its icosahedral shape, and the internal genome was also confirmed to be slowly leaking out (22).

Furthermore, investigation of the influence of storage conditions on the antiviral activity of vacuoles revealed interesting results. We confirmed that the antiviral effect of the extracted vacuoles was improved by storing them at 4°C, and we compared and verified the antiviral effects of vacuoles under different storage conditions. The antiviral effect of vacuoles stored even briefly at 4°C increased compared to vacuoles that were extracted and used immediately. In particular, vacuoles stored in pellet state without resuspension in dH_2_O had improved antiviral activity compared to the original vacuoles. The results of virus titer measurement using plaque assay showed that the titer of the T4 virus treated with vacuoles stored in a pellet state decreased further. These results are considered due to changes in the activity of the vacuolar enzyme caused by storage. Analysis of the soluble fraction of yeast vacuolar lumen identified approximately 260 polypeptides, the majority of which were found to be vacuolar proteases (19). Previous studies have reported that, among the numerous hydrolytic enzymes, the activities of cathepsin B and cathepsin D—homologs of proteinase A, which are abundantly found in vacuoles—increase with storage time (32). Contrary to previous reports suggesting that inhibition of cathepsins is beneficial for antiviral effects, this paradoxical finding indicates that, in addition to cathepsins involved in viral infection and entry, the roles of various other hydrolytic enzymes likely act in a complex and significant manner, warranting further research (33, 34). Additionally, by confirming through morphological analysis that the size increases and surface charge properties change, we find that structural change of the vacuolar membrane occurs when stored in the pellet state. As the zeta potential value of the nanoparticles converges to 0 due to changes in the vacuolar membrane surface charge properties, it is expected that non-specific interaction between the T4 virus and the vacuoles is induced and attachment with more vacuoles occurs (26). Furthermore, vacuoles stored in pellet state have a low pH of approximately 5.8 (the original vacuoles have a pH of approximately 6.4), which also supports the finding that bacteriophages are inactivated at low pH. This result is hypothesized to be due to competitive exclusion interactions in which increasing H^+^ at low pH competes with Ca^2+^ or other bivalent cations at some sites on the bacteriophage surface, changing the overall charge distribution and causing the bacteriophage to lose stability and become inactive (35).

Overall, our preliminary findings highlight the potential of vacuoles extracted from yeast as promising antiviral agents against non-enveloped tailed viruses. The antiviral activity and morphological changes observed due to vacuole treatment provide insight into the mechanism of antiviral action. Furthermore, optimization of vacuoles’ storage conditions can further improve the activity, paving the way for a variety of applications in combating viral infections. However, further studies are needed to explore the underlying mechanisms responsible for the observed antiviral activity, exploring the interaction between vacuole-viral particles at the molecular level and evaluating the effect on other non-enveloped viruses. This study is the first step toward developing an understanding of vacuoles as eco-friendly antiviral agents, providing new strategies for virus control/prevention, and addressing the current global health challenges for viral infectious diseases.

## Data Availability

All data generated or analyzed during this study are included in this published article and its supplemental material.

## SUPPLEMENTAL MATERIAL

Supplemental material spectrum.02886-25-s0001.docx Fig. S1 and S2; Tables S1 and S2.

ASM does not own the copyrights to Supplemental Material that may be linked to, or accessed through, an article. The authors have granted ASM a non-exclusive, world-wide license to publish the Supplemental Material files. Please contact the corresponding author directly for reuse.
