# Supplementary material for: Storage conditions and antiviral efficacy of yeast-derived vacuoles on T4 virus
Source: Microbiol Spectr. 2026 Feb 26;14(4):e02886-25. doi: 10.1128/spectrum.02886-25 (PMC13055312; doi:10.1128/spectrum.02886-25)
Supplement: Supplemental material — Fig. S1 and S2; Tables S1 and S2. [file spectrum.02886-25-s0001.docx]

**Supplementary Information (SI)**

**Storage Conditions and Antiviral Efficacy of Yeast-Derived Vacuoles on T4 Virus**

**Short title: Storage and efficacy of vacuoles against T4 virus**

Taehwan Kim^1,2^, Jae-Hwang Jeong^3^, Yang-Hoon Kim^4,*^, and Jiho Min^1,*^

^1^Graduate School of Semiconductor and Chemical Engineering, Jeonbuk National University, 567 Baekje-daero, Deokjin-gu, Jeonju-si, Jeonbuk State, 54896, Republic of Korea

^2^Research Division for Biotechnology, Advanced Radiation Technology Institute, Korea Atomic Energy Research Institute (KAERI), Jeongeup, Jeonbuk State, 56212, Republic of Korea

^3^Biopharmaceutical Engineering, Chungbuk Provincial University, 194-31, Osongsaengmyeong 1-ro, Osong-eup, Heungdeok-Gu, Cheongju, Chungbuk, 29046, Republic of Korea

^4^School of Biological Science, Chungbuk National University, Chungdae-ro 1, Seowon-Gu, Cheongju, Chungbuk, 28644, Republic of Korea

***Corresponding Author:**

(Phone) +82-63-270-2436

(Fax) +82-63-270-2306

(E-mail) jihomin@jbnu.ac.kr

**^*^Co-corresponding Author**

(Phone) +82-43-261-3575

(Fax) +82-43-267-9600

(E-mail) kyh@chungbuk.ac.kr

This supplementary information includes Number of figures: 2; Number of tables: 2; Number of pages: 7.

**Submitted to *Microbiology Spectrum***

**This supplementary information includes:**

**S1. Supplementary Figures**

**S2. Supplementary Tables**

**SUPPLEMENTARY FIGURE LEGENDS**

**Supplementary Figure S1. Antiviral effect of freshly extracted vacuoles.**

Viral titers of *Escherichia virus T4* versus vacuole concentration. The vacuoles were used immediately after extraction. Final concentrations were adjusted to 25, 50, and 100 μg/mL using distilled water (dH_2_O). The data were analyzed using Sigma Plot 14 (Systat Software Inc., USA), and a p value < 0.1 was considered significant. Data are expressed as the (means of the infection inhibition rate ± SD) in %.

**Supplementary Figure S2. Changes in structural size.**

The structural size change was confirmed using DLS. The data were analyzed using Sigma Plot 14 (Systat Software Inc., USA), and a *p* value < 0.05 and a *p* value < 0.01 were considered significant. Data are expressed as the (means of the size of vacuoles ± SD) in nm. **P* < 0.05 vs. original vacuoles. ***P* < 0.01 vs. original vacuoles.

**SUPPLEMENTARY TABLE LEGENDS**

**Supplementary Table S1. Antivirus activity of vacuole treatment against T4 virus**

**Supplementary Table S2. Enhanced antivirus activity of vacuole treatment against T4 virus with storage condition**

*, *P* < 0.05 and **, *P* < 0.01 (significantly different from original vacuoles).


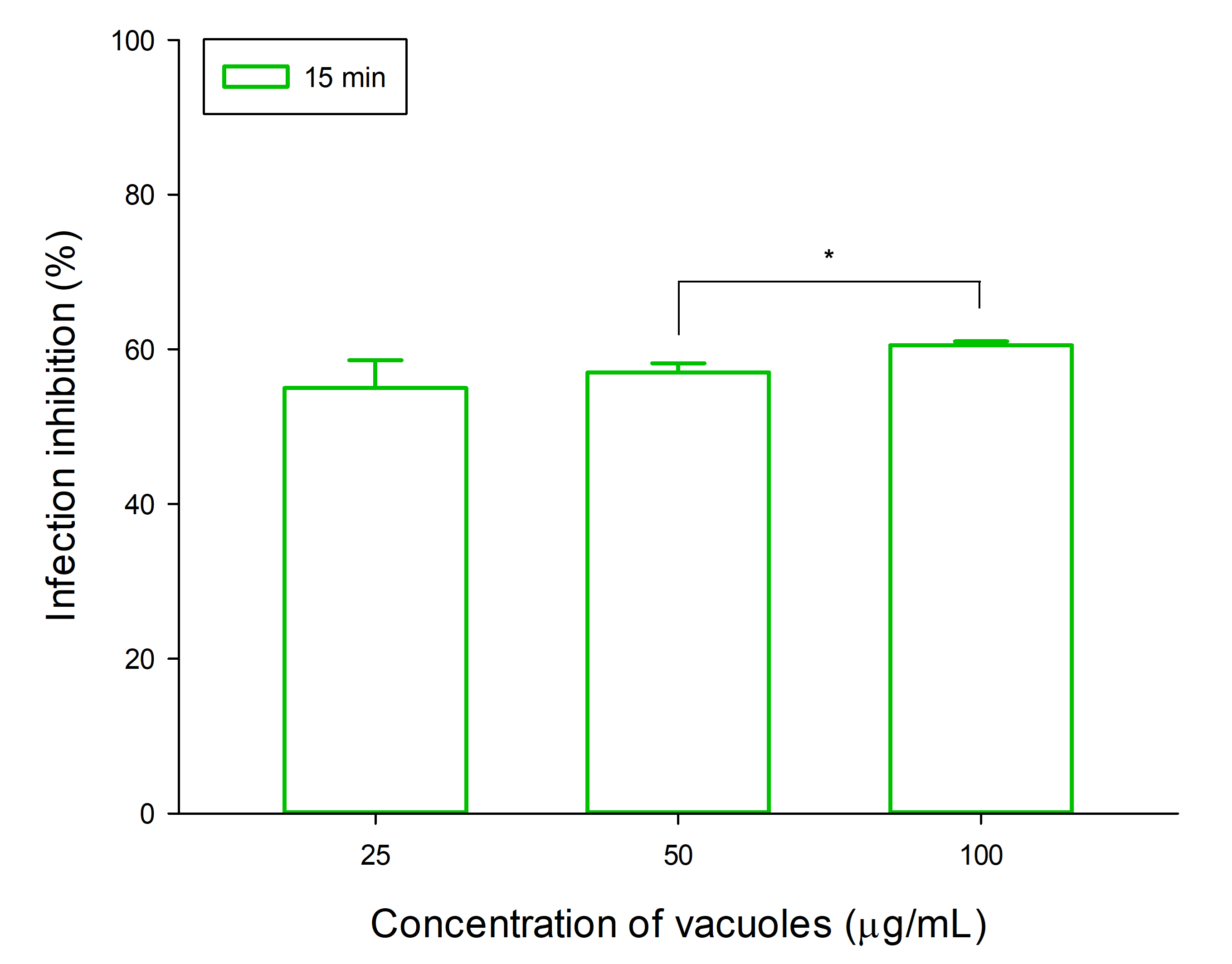


**Supplementary Figure S1.**


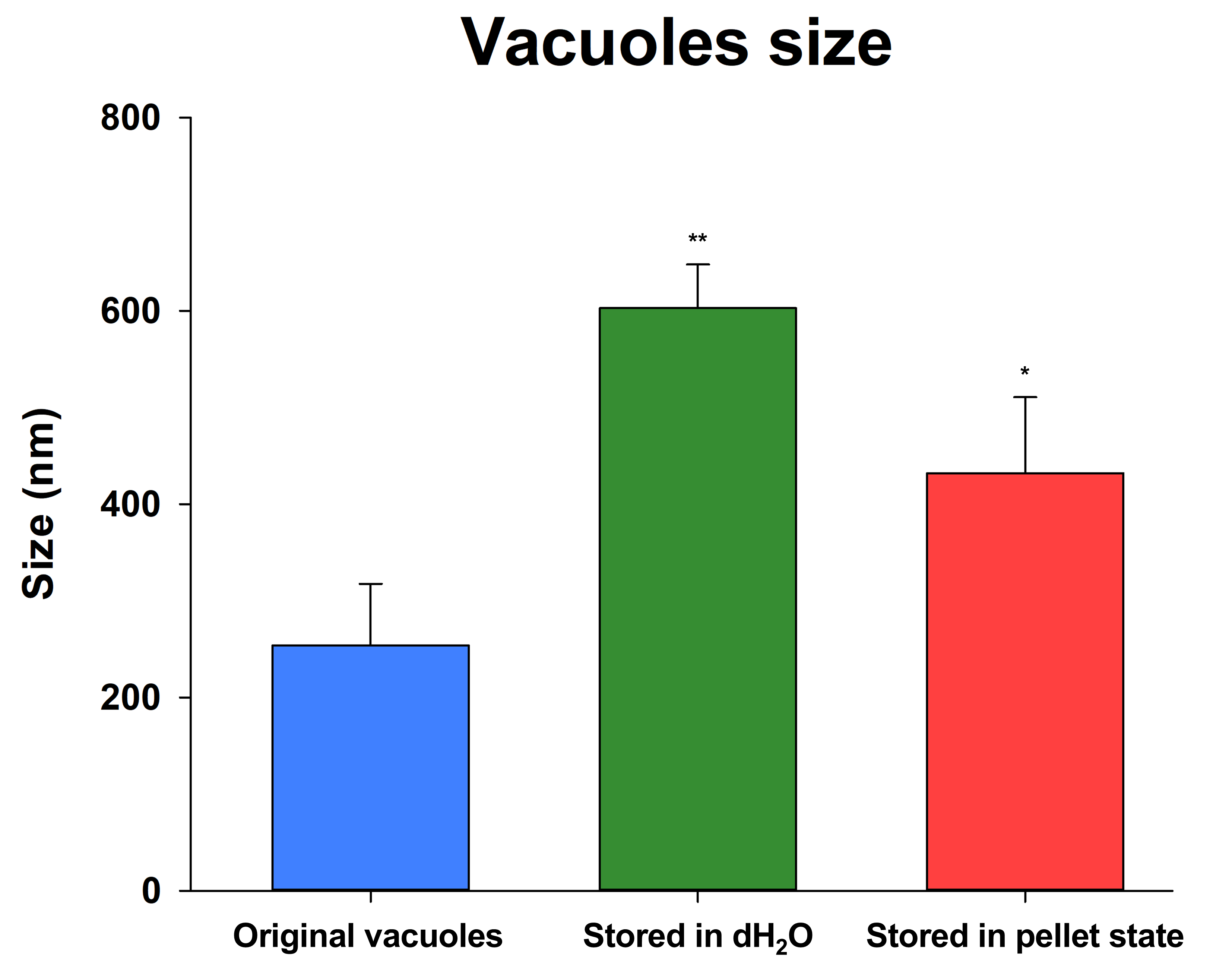


**Supplementary Figure S2.**

**Supplementary Table S1. Antivirus activity of vacuole treatment against T4 virus**

| **Concentration of vacuoles**  **(μg/mL)** | **Exposure time (min)** | **Treatment**  **Log(pfu/mL) (±SD)** | **Antiviral effect (%)** |
| --- | --- | --- | --- |
| 50 | 15 | 5.12 (±0.08) | 72.20% |
|  | 30 | 5.09 (±0.07) | 73.81% |
|  | 60 | 4.88 (±0.06) | 84.40% |
|  | 120 | 5.03 (±0.07) | 77.66% |
| 250 | 15 | 4.86 (±0.12) | 84.52% |
|  | 30 | 4.80 (±0.14) | 86.16% |
|  | 60 | 4.80 (±0.13) | 86.32% |
|  | 120 | 4.77 (±0.11) | 87.32% |

**Supplementary Table S2. Enhanced antivirus activity of vacuole treatment against T4 virus with storage condition**

| **Concentration of vacuoles (μg/mL)** | **Exposure time (min)** | **Storage condition** | **Antiviral effect (%)** | **Increased antiviral effect compared to original vacuoles** |
| --- | --- | --- | --- | --- |
| 25 | 1 | Original vacuoles | 41.42 ± 2.94 | 26.44^**^ |
|  |  | Stored in pellet state | 67.86 ± 3.89 |  |
|  | 5 | Original vacuoles | 55.11 ± 9.57 | 11.63 |
|  |  | Stored in pellet state | 66.75 ± 7.25 |  |
|  | 15 | Original vacuoles | 60.24 ± 7.51 | 25.37^*^ |
|  |  | Stored in pellet state | 85.62 ± 0.81 |  |
| 50 | 1 | Original vacuoles | 39.94 ± 2.59 | 28.90^*^ |
|  |  | Stored in pellet state | 68.84 ± 10.84 |  |
|  | 5 | Original vacuoles | 47.02 ± 11.02 | 26.80 |
|  |  | Stored in pellet state | 73.82 ± 2.39 |  |
|  | 15 | Original vacuoles | 69.07 ± 2.66 | 4.28 |
|  |  | Stored in pellet state | 73.35 ± 2.62 |  |
| 100 | 1 | Original vacuoles | 54.45 ± 4.45 | 10.25 |
|  |  | Stored in pellet state | 64.70 ± 12.52 |  |
|  | 5 | Original vacuoles | 57.55 ± 1.63 | 15.56^**^ |
|  |  | Stored in pellet state | 73.11 ± 3.04 |  |
|  | 15 | Original vacuoles | 70.12 ± 5.14 | 1.89 |
|  |  | Stored in pellet state | 72.01 ± 8.37 |  |
| *, *P* < 0.05 and **, *P* < 0.01 (significantly different from original vacuoles). | | | | |
